# Effects of Group Size on Behavior, Reproduction, and mRNA Expression in Brains of Brandt’s Voles

**DOI:** 10.3390/brainsci13020311

**Published:** 2023-02-12

**Authors:** Wei Lu, Shuli Huang, Jing Liu, Erdenetuya Batsuren, Guoliang Li, Xinru Wan, Jidong Zhao, Zuoxin Wang, Wenxuan Han, Zhibin Zhang

**Affiliations:** 1 State Key Laboratory of Integrated Management of Pest Insects and Rodents, Institute of Zoology, Chinese Academy of Sciences, Beijing 100101, China; 2 University of Chinese Academy of Sciences, Beijing 100049, China; 3 Institute of Plant Protection, Ulaanbaatar 17024, Mongolia; 4 Department of Psychology, Florida State University, Tallahassee, FL 32306, USA; 5 College of Resources and Environmental Sciences, China Agricultural University, Beijing 100193, China; 6 CAS Center for Excellence in Biotic Interactions, University of Chinese Academy of Sciences, Beijing 100049, China

**Keywords:** Brandt’s voles, gene expression, group size, reproduction, stress

## Abstract

For social animals, a moderate group size is greatly important to maintain their reproductive success. However, the underlying neurobiological mechanism of group size on behavior and reproduction has rarely been investigated. In this study, we examined the effects of group size (1, 2, 4 pairs of adult male and female voles raised per cage) on behavior and reproduction. Meanwhile, the mRNA expression of stress and reproduction response-related genes in male brains was detected. We found that Brandt’s voles (*Lasiopodomys brandtii*) in the large-sized group fight more severely than those in the small-sized group. Meanwhile, male voles were more anxious than females. The average number of embryos and litters per female in the medium-sized group was significantly higher than that of large-sized group. In male voles, stress- or reproduction-response mRNA expressions were more related to final group size or final density due to death caused by fighting. Our results indicated that a moderate group size was beneficial to the reproductive output of Brandt’s voles. Our study highlights the combined effects of stress- or reproduction-related gene expression or behavior in regulating the fitness of voles with different group sizes.

## 1. Introduction

Group living can decrease the predation risk of animals, increase the opportunity to gain food resources, and enhance thermoregulation, but it also can inhibit reproduction, increase the risk of infection by parasites, and exacerbate competing pressure for resources [1,2,3,4]. Animals living in groups experience many kinds of social interaction, for example, social recognition and social memory and interactions with peers, competitors, possible paired partners, and offspring [5]. Aggressive behavior would increase with the increase in group size, so as to gain and maintain resources [6,7]. Mice experiencing social-defeat/overcrowding and chronic subordinate colony housing for 19 days both exhibit elevated anxiety levels [8]. It is detrimental for animals when the group size is too small or too big, so a moderate group size is beneficial for reproduction and the survival of the population [2]. Female prairie voles (*Microtus ochrogaster*) living in groups of three adults have the highest reproductive success (the number of young survive to 12 or 30 days per adult female in a group). Furthermore, female voles living with two adult males reproduce more successfully than those living in groups with fewer (0–1 male) or more males (3–6 males) [4]. The lifetime reproductive success of male Alpine marmots (*Marmota marmota*) increases until the average group size reaches 5.66, and in females that number is 7.29. Male fitness was maximized when existing in an optimal group size [9]. Wrangham proposed the hypothesis that primate individuals in a small group have disadvantages in the competition between groups, while the individuals in the large group compete fiercely with group members, so the relationship between the birth rate of primate offspring and group size is an inverted-U curve [10]. Zhang proposed a dome-shaped density-dependency model, in which cooperation of animals occurs in low-density conditions but competition occurs in high-density conditions. Although the dome-shaped effects of group size or density on reproduction and the survival of animals have been investigated, the underlying neurobiological mechanisms remain unclear [11].

Continuous stress has severe long-lasting adverse effects on brain function and behavior. Social stress activates the HPA axis. Corticotropin-releasing hormone (CRH) is released from the hypothalamus into the pituitary portal system, which stimulates anterior pituitary cells to release adrenocorticotropic hormone (ACTH), and then glucocorticoids (GCs) are released from the adrenal cortex stimulated by ACTH [12,13]. GCs exert negative feedback regulation by inhibiting the encoding of the pro-opiomelanocortin (*POMC*) gene, directly inhibiting the release of ACTH to the peripheral system or reducing the binding of CRH-Receptor 1 [14,15]. Acute social defeat increases plasma adrenocorticotrophic hormone and corticosterone (CORT) levels and enhances *CRH* mRNA in the paraventricular nucleus (PVN) brain area of NMRI mice [16]. In aggression tests, adult oxytocin (*OT*)-KO males show more aggressive behavior than WT controls [17]. Intracerebroventricular (ICV) injection of OT will not only reduce the level of circulating CORT, but also reduce the level of ACTH in rats and mice after stress [18]. The biting attacks of resident male hamsters on intruders decrease significantly when they are injected with arginine vasopressin (AVP) antagonist into the anterior hypothalamus (AHA) [19]. After weaning, social isolation increases AVP-immunoreactivity in the supraoptic nucleus (SON) compared to housing with siblings in juvenile females [20]. Aggression is reduced, by increasing 5-HT1A activity [21].

Animal reproduction is regulated by the hypothalamus–pituitary–gonadal (HPG) axis. The feedback signal from sex hormones can modulate gonadotropin-releasing hormone (GnRH) and gonadotrophin secretion. Mammalian sexual maturity is induced by pulse secretion of GnRH in the medial preoptic area (MPOA) [22]. In an unstable group-living environment (ascending a social hierarchy), the level of *GnRH* mRNA of CD1 mice in MPOA elevates [23]. Kisspeptin (coded by the *Kiss1* gene), whose neurons are mainly located in the arcuate nucleus (Arc) area, activates the pulse secretion of GnRH in this area [24] and modulates gonadotropin secretion, the onset of puberty, and control of fertility. [25]. *Kiss1* receptor-knockout male mice cannot exhibit mating behavior such as mounting, thrusting, or ejaculating [26]. In recent years, it has been found that RFamide-related peptide (Rfrp) can inhibit the secretion of GnRH and can also reduce the secretion of luteinizing hormone (LH). Rfrp in mammals is produced by a precursor peptide, which is cleaved into two active peptides: Rfrp1 and Rfrp3 [27]. Rfrp-3 inhibits the sexual maturation of subordinate female rats, suggesting that Rfrp plays a role in regulating reproductive disorders induced by social stress [28]. Testosterone signals in the male brain are either directly through the androgen receptor (Ar) or indirectly through the estrogen receptor (Er) following aromatization into 17-β-estradiol (E2) [29]. However, the negative feedback of sex hormones does not directly target GnRH neurons, but rather Kisspeptin neurons, because the GnRH neurons lack Ar and Erα [30]. In the adult rat brain, *Ar* and *Esr1* mRNA-expressing neurons are widely distributed in the hypothalamus, which is thought to mediate the hormonal control of copulatory behavior as well as the neural control of gonadotropin release, and in the telencephalon, which provides strong inputs to the hypothalamus [31]. Erα immunoreactive cells in the MPOA and bed nucleus of the stria terminalis (BNST) area of female prairie voles isolated for 21 days are significantly fewer than those of isolated males and those of housed with stranger females [32]. Early social stress makes male guinea pigs (*Cavia aperea f. porcellus*) express fewer Ars in the MPOA and Arc areas compared with unexperienced control males [33]. The above neuropeptides play an important role in modulating social behavior and reproduction. In this paper, we aimed to explore the effect of group size on the expression of these neuropeptides in brains of Brandt’s voles (*Lasiopodomys brandtii*).

Brandt’s voles are widely distributed in the Inner Mongolia grassland of China, the central and eastern part of Mongolia, and the Baikal Lake region of Russia [34,35]. They live in groups, and each group occupies a burrow system which is 50–100 m^2^ and 25 m in diameter [36,37]. There are 2–24 voles per group in nature, which includes 2 to 7 breeders based on analysis using genetic markers. Most groups of voles are made up of close relatives. Their mating system is promiscuous [38,39]. Brandt’s voles breed seasonally, mainly from April to August, and their group structure shows strong seasonality. In the early spring, the number of members in one group decreases gradually. Other members die or migrate to new breeding groups due to mating competition. Overwintering adult males and females form a family group for breeding. Newborn offspring will disperse to build new families when they become adults, but most do not breed in the year of birth. When the autumn comes, voles begin to cluster in preparation for overwintering. They have a tendency to select homologous individuals when clustering. The social behaviors of Brandt’s voles are influenced by sex, social hierarchy, reproductive status, and season [39]. The females often give birth to 2–4 litters annually, and the litter size varies greatly, ranging from 2 to 15 [34]. The gestation and weaning period are both around 21 days [40]. Huang et al. found that the Brandt’s voles living in high-density groups (4 males and 4 females) attacked each other more intensely; AVP expression increased, while OT expression reduced in the MPOA, PVN, and amygdala region [41]. Male voles living in high density (five male voles per cage) had more aggressive behavior, elevated serum corticosterone, increased spleen weight, and lower mRNA levels of tryptophan hydroxylase 2 (*TPH2*), *Htr1a*, and 5-hydroxytryptamine receptor 1B (*Htr1b*) in the amygdala and the dorsal raphe nucleus (DRN) than those living in low density (three male voles per cage) [42]. The available studies above are all based on the density of Brandt’s voles. Higher population densities are accompanied by increased group size and decreased housing space. So far, the effects of group size on reproduction and anxiety of Brandt’s voles, especially the neuromodulatory mechanism, have not been studied.

In this study, we created a circumstance to examine the effects of group size (1, 2, 4 pairs of adult male and female voles per cage) on the behavior, reproduction, and survival of Brandt’s voles as well as the underlying neurobiological regulation mechanism. This study aimed to test the predictions that (1) voles in moderate-sized groups would have the highest reproduction performance (average reproductivity per female, i.e., average litter size per female) and mRNA expression of reproduction-related genes (*GnRH*, *Kiss1*, *Esr1*, and *Ar*, but not *Rfrp*), but moderate mortality, anxiety behavior, and mRNA expression of stress-related genes (*CRH*, *AVP*, *POMC*, *OT*, and *Htr1a*); (2) voles in large-sized groups would have the highest mortality, anxiety behavior, and mRNA expression of stress-related genes, but lowest reproduction performance and mRNA expression of reproduction-related genes; (3) voles in small-sized groups would have the lowest mortality, anxiety behavior, and mRNA expression of stress genes, as well as a lower average reproductivity per female. 

## 2. Materials and Methods

### 2.1. Animals

The experimental Brandt’s voles were captured by live traps from the grassland of Dongwuqi, Inner Mongolia, China in 2018. They were transported to laboratory in the Maodeng pasture land. The male and female voles were raised in separated cages for adaptation for two weeks. The cage size was 30 × 20 × 30 cm^3^ (L × W × H) and there were 3–5 voles in each cage. Brandt’s voles weighing 25–35 g were chosen for conducting the following experiments for 35 successive days. The light was natural from August to October. The room temperature was between 10 to 22 °C from August to mid-September, and room temperature was artificially controlled to 22 °C with electric heaters from late September to early October. Rabbit chow and water were supplied ad libitum, and the bedding was changed once a week.

### 2.2. Experimental Design

**Figure 1:**
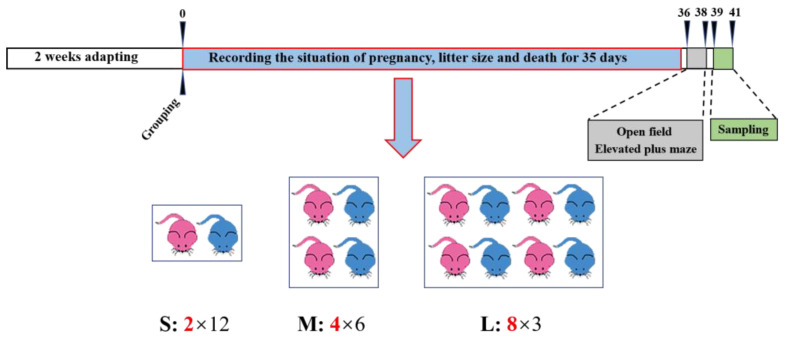
Schematic diagram of the experimental design and procedures. After two weeks’ adaption, voles were randomly assigned into one of the three experimental groups. The small group (S) contained 1 male and 1 female in a small-sized plastic box (L × W × H = 26 × 15 × 14 cm^3^), the medium group (M) contained 2 males and 2 females in a medium-sized plastic box (L × W × H = 30 × 26 × 14 cm^3^), and the large group (L) contained 4 males and 4 females in a large-sized plastic box (L × W × H = 52 × 30 × 14 cm^3^). There were 12 replications for the S group, 6 replications for the M group, and 3 replications for the L group. Food and water were provided ad libitum daily to each box. Females’ pregnancy as well as litter size and death were checked daily throughout the 35-day treatment period. At the end of the experiment, there were 24 voles (12 males and 12 females) in the S group, 22 voles (15 males and 7 females) in the M group, and 14 voles (7 males and 7 females) in the L group. Thereafter, subjects went through the open field and elevated plus maze tests on day 36–38, and brain tissues of male voles were harvested on day 39–41 for subsequent mRNA analyses. Pink voles were females, and blue voles were males.

After two weeks of acclimating (Figure 1), voles were randomly assigned into one of three experimental groups with different group sizes (each vole occupied a same space). For the small-sized group (S), 2 voles (1 male and 1 female) were placed in a small-sized plastic box (26 × 15 × 14 cm^3^), which had 12 replications. For the medium-sized group (M), 4 voles (2 males and 2 females) were placed in a middle-sized plastic box (30 × 26 × 14 cm^3^), which had six replications. For the large-sized group (L), 8 voles (4 males and 4 females) were placed in a large-sized box (52 × 30 × 14 cm^3^), which had three replications. We marked voles using a numbered animal ID (size: 1.4 × 8 mm^2^, Beijing Raybaca Technology Co., Ltd., Beijing, China) underneath their back skin. One female in the M group and one in the L group had pups born. The new pups and dead individuals were checked and taken away daily throughout the 35-day treatment period, during which food and water were provided ad libitum. It should be noted that the sizes of the boxes were designed so that each vole could have equal space and the setting density of each group (2 voles/unit area; the bottom area of small box was defined as unit area) was the same. The final numbers of males and females in the M and L groups were not in line with initial setting, because the sex of a few voles was identified incorrectly and 12 voles died as a result of aggression. On days 36–38, voles’ anxiety-like behaviors (S group, 12 males and 12 females; M group, 15 males and 7 females; L group, 7 males and 7 females) were tested in the morning using the open field test as well as in the afternoon using the elevated plus maze, with 4–5 hr intervals between the two tests. On days 39–41, all subjects were sacrificed, and brain samples of male voles were collected for the subsequent gene analyses. The final group size is shown in Table S1. Because mortality of voles changed the final group size, we excluded two replications (one in M group and one in L group) with final number of 2 voles to analyze the difference in the mRNA expression and behavior test of last observation between the S, M, and L groups.

### 2.3. Behavior Observations

Animal interactions in each box were recorded using video cameras (Canon, Tokyo, Japan) for a total of 1.5 hrs (0730–0800, 1230–1300, and 1730–1800, respectively [43,44]) on Day 1 and 35. We recorded the animal interactions in all the boxes at the same time. Four replications of the S group were covered by one camera, two replications of the M group were covered by one camera, and one replication of the L group were covered by one camera. So, there were 9 cameras used to record the behaviors. The frequency and duration of aggressive behaviors (e.g., fighting: forelimb fighting and head shoving; chasing: continuous and fast catching up; biting: use of teeth on skin) towards the conspecifics were quantified [45,46,47], and the means were calculated for each box.

Anxiety-like behaviors were tested in the open field tests and elevated plus maze, respectively, using previously established methods [48]. The open field box (L × W × H = 50 × 50 × 30 cm^3^) was made of white plastic, and its bottom was divided into three areas: central area, middle area, and edge area. The size of the central area was L × W = 20 × 20 cm^2^, the edge area was 7 cm along the edge, and the remaining area was the middle area. The boundaries of each area were drawn in black. The behavioral test was conducted for 5 min, during which anxiety behaviors (the duration and frequency of the subject in the central area) and locomotion (the total distance traveled by the subject in the field) were recorded. In the open field, voles making fewer entries to or spending less time in the center area were defined as more anxious. The box was cleaned with 75% alcohol between animals. At the end of the experiment, 24 voles (12 males and 12 females) in the S group, 20 voles (14 males and 6 females) in the M group, and 12 voles (6 males and 6 females) in the L group were analyzed.

The elevated plus maze was made of glass, including two open arms (44.5 × 11.5 cm^2^) and two closed arms (44.5 × 11.5 × 15 cm^3^) (the two parts were perpendicular to each other to form a cross); all were attached with a central platform (11.5 × 11.5 cm^2^). The maze was elevated 52 cm above the ground. The test was started by placing a vole on the central platform with its head facing a closed arm. In the elevated plus maze, voles having fewer entries to or spending less time in open arms were defined as more anxious. The duration of the test was 5 min. The frequency of entering the open arms and the duration spent in open arms were recorded. The maze was cleaned with 75% alcohol between animals. A total of 13 male voles (S group, *n* = 5, M group, *n* = 6, L group, *n* = 2) and 7 female voles (S group, *n* = 4, M group, *n* = 3) were excluded as falling to the ground from elevated plus maze during recording. So, 15 voles (7 males and 8 females) in the S group, 13 voles (9 males and 4 females) in the M group and 10 voles (4 males and 6 females) in the L group were analyzed. All videos were analyzed using EthoVision XT11.5 software (Noldus, Wageningen, Netherlands). To minimize observer bias, blinded methods were used when all behavioral data were quantified.

### 2.4. Sampling

Considering that the 7-day estrus cycle of female voles would affect gene expression in brain [49], we just used the males for analyzing gene expression. Male subjects (*n* = 7, 6, and 6, respectively, in the S, M, and L groups) were sacrificed at 0700–1000 one day after their behavior tests. Subjects were anesthetized with an intraperitoneal injection of 0.5% sodium pentobarbital. Their brains were taken quickly and frozen on dry ice, then stored in liquid nitrogen for subsequent analysis of the gene expression. Female voles (*n* = 12, 7, and 7, respectively, in the S, M, and L groups) were also sacrificed to check their pregnancy status and the number of embryos.

### 2.5. Reproductivity and Survival Rate

If vaginal plugs, or embryos in uterus or litter birth of females, were observed, we defined these females as the pregnant individuals. The proportion of pregnant females was defined as the pregnancy rate. The average number of embryos and litters per pregnant female used to define the average litter size. The average number of embryos and litters per female was defined as the average reproductivity per female. The proportion of surviving individuals by the end of the experiment was defined as the survival rate.

### 2.6. RNA Isolation and Reverse Transcription-PCR

To study the impact of group size on gene expression, brains from male subjects were processed for RT-PCR. We focused on *OT*, *AVP*, *CRH*, *POMC*, *GnRH*, *Kiss1*, *Rfrp*, *Htr1a*, *Esr1*, and *Ar*. Frozen brains were sectioned coronally at 200 μm. Tissue punches were taken from the MPOA, PVN, AHA, dorsomedial hypothalamus (DMH), Arc, and medial amygdala (MeA) areas, which were the main distribution and functional areas associated with these genes [22,24,27,31,32,50]. Tissue punches were transferred to 1.5 mL RNase-free centrifuge tubes. Total RNA was extracted by Trizol reagent (Invitrogen, Waltham, USA). cDNA was synthesized using a reverse transcriptase kit (Thermo Fisher Scientific, #K1622, Waltham, USA), following the manufacturer’s protocol. The primers (Table 1) were designed using NCBI Primer BLAST software and relative expression of genes was performed using SYBR Green PCR kit (Thermo Fisher Scientific, #K0223, Waltham, USA), following the manufacturer’s instruction. Glyceraldehyde-3-phosphate dehydrogenase (*Gapdh*) was used to normalize the gene expression level [41]. The data were analyzed by the 2^−ΔΔCt^ method. Based on previous studies, we defined *CRH*, *POMC*, *AVP*, *OT*, and *Htr1a* as the stress-response genes and *GnRH*, *Kiss1*, *Rfrp*, *Esr1*, and *Ar* as the reproduction-response genes in view of their involved primary function, although each set of genes may affect the other function directly as well as indirectly.

### 2.7. Statistical Analysis

In order to avoid the overuse of voles, the data of small-sized groups were shared with another experiment that was submitted to another journal. Data of aggression behavior were analyzed using one-way analysis of covariance (ANCOVA), with the proportion of male voles as a covariate. Post hoc comparisons were performed with Bonferroni’s correction. Considering the effect of group size, sex, and the potential role of interaction between group size and sex on the anxiety behavior of voles, linear mixed model (LMM) analysis was performed for the data from the open field test and elevated plus maze, followed by TukeyHSD test. The cage ID was defined as a random factor.

Data of survival rate and female pregnancy rate were analyzed using a general linear mixed model (GLMM) with group size as the fixed effect and cage ID as a random factor. Data of average litter size, average reproductivity and mRNA expression were analyzed by LMM. Group size was defined as fixed factor and cage ID was defined as random factor. Because a few animals died, which ultimately changes the group size and final density, Spearman correlation analysis was used to examine the correlation between gene expression and final group size or final density. The data were analyzed by R software (4.2.1). Graphs were illustrated with Graphpad software (version: 6).

## 3. Results

### 3.1. Impacts of Initial Groups Size on Aggression Behaviors and Anxiety Behaviors

**Figure 2:**
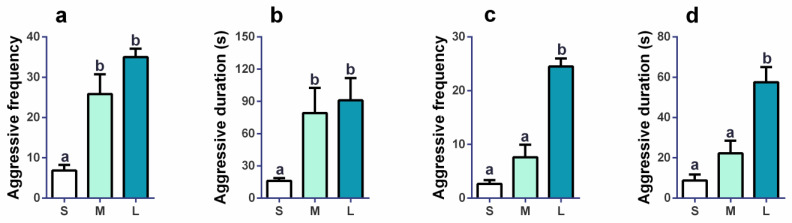
The aggression behavior of voles on day 1 and day 35. (**a**) The aggressive frequency on day 1. (**b**) The aggressive duration on day 1. *n* = 12 (S group), 6 (M group), and 3 (L group) for (**a**,**b**). (**c**) The aggressive frequency on day 35. (**d**) The aggressive duration on day 35. One-way ANCOVA was performed, followed by post hoc comparisons with Bonferroni’s correction. *n* = 12 (S group), 5 (M group), and 2 (L group) for (**c**,**d**). S, M, and L are small group, medium group, and large group, respectively. Different letters mean that *p* values are statistically significant. Bars indicate mean ± SEM.

We observed the aggression behaviors of Brandt’s voles in each group at the initial phase and last phase of this experiment and counted the aggressive frequencies and duration over one day (0.5 h in the morning, at noon, and in the evening individually). On day 1, aggressive frequency (Figure 2a, *F*_2,17_ = 22.306, *p* < 0.001) and duration (Figure 2b, *F*_2,17_ = 8.893, *p* = 0.002) were both significantly different among the three groups. Compared to the S group, the frequency of aggression was 3.78- and 5.12-fold higher in the M (*p* < 0.001) and L groups (*p* < 0.001). The duration of aggression in the S group was 4.92- and 5.66-fold lower in the M (*p* = 0.020) and L groups (*p* = 0.010). On day 35, aggressive frequency (Figure 2c, *F*_2,15_ = 52.627, *p* < 0.001) and duration (Figure 2d, *F*_2,15_ = 24.421, *p* < 0.001) were both significantly different among the three groups. Compared to the L group, the frequency of aggression was 3.22- and 9.18-fold lower in the M (*p* < 0.001) and S groups (*p* < 0.001), respectively. The duration of aggression in the L group was 2.59- and 6.57-fold higher than in the M (*p* < 0.001) and S groups (*p* < 0.001). The results indicated that the aggression in the L groups was more severe in the L group than in the S group.

**Figure 3:**
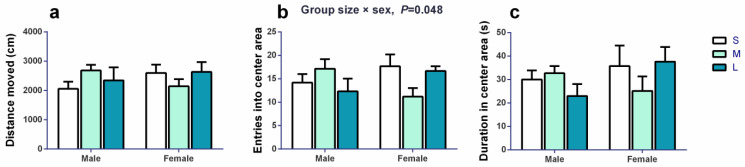
The activity of voles in the open field on day 36–38. (**a**) Overall locomotor activity was not affected by group size, sex, or the interaction between them. (**b**) Entries into central area were not significantly affected by group size or sex, but were significantly affected by the interaction between group size and sex. (**c**) Duration in the central area was not significantly affected by group size, sex, or the interaction between them. Linear mixed model (LMM) analysis was performed with group size and sex as the fixed effect, followed by TukeyHSD test. The cage ID was defined as a random factor. *n* = 24 (S group: 12♂12♀), 20 (M group: 14♂6♀) and 12 (L group: 6♂6♀) for all figures. S, M, and L are small group, medium group, and large group, respectively. Bars indicate mean ± SEM. Bars with different letters differ significantly from each other.

In the open field test, the distance moved (Figure 3a) was not significantly affected by group size (*F*_2,11_ = 0.183, *p* = 0.836), sex (*F*_1,41_ = 0.123, *p* = 0.728) or the interaction between them (*F*_2,41_ = 2.244, *p* = 0.119). The frequency of entering the center area (Figure 3b) was not significantly affected by group size (*F*_2,56_ = 0.410, *p* = 0.666) or sex (*F*_1,56_ = 0.109, *p* = 0.742), but it was significantly affected by the interaction between group size or sex (*F*_2,56_ = 3.198, *p* = 0.048). Duration in the center area (Figure 3c) was not significantly affected by group size (*F*_2,6_ = 0.208, *p* = 0.818), sex (*F*_1,29_ = 0.924, *p* = 0.345), or the interaction between group size and sex (*F*_2,29_ = 1.689, *p* = 0.202).

**Figure 4:**
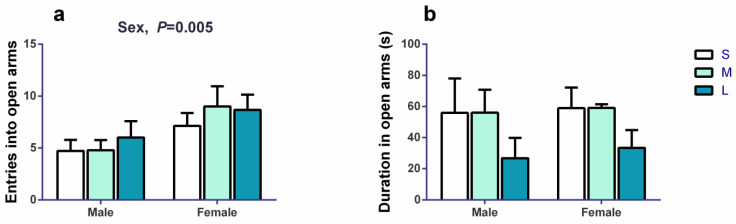
The activity of voles in the elevated plus maze on day 36–38. (**a**) Entries into open arms were significantly affected by sex, and were higher in females than in males, but were not significantly affected by group size or the interaction between sex and group size. (**b**) Duration in open arms was not significantly affected by group size, sex, or the interaction between them. Linear mixed model (LMM) analysis was performed with group size and sex as the fixed effect, followed by TukeyHSD test. The cage ID was defined as a random factor. *n* = 15 (S group: 7♂8♀), 13 (M group: 9♂4♀), and 10 (L group: 4♂6♀) for all figures. S, M, and L are small group, medium group, and large group, respectively. Bars indicate mean ± SEM.

In the elevated plus maze, the frequency of entries into open arms (Figure 4a) was significantly affected by sex (*F*_1,38_ = 9.001, *p* = 0.005), and it was 1.61-fold higher in females than in males, but it was not affected by group size (*F*_2,38_ = 0.707, *p* = 0.499) or the interaction between group size and sex (*F*_2,38_ = 0.309, *p* = 0.736). The duration in open arms (Figure 4b) was not significantly affected by group size (*F*_2,5_ = 0.956, *p* = 0.444), sex (*F*_1,20_ = 0.094, *p* = 0.762), or the interaction between group size and sex (*F*_2,20_ = 0.036, *p* = 0.965). The results indicated that the male voles might be more anxious.

### 3.2. Effects of Initial Group Size on Survival Rate and Reproductivity

**Figure 5:**
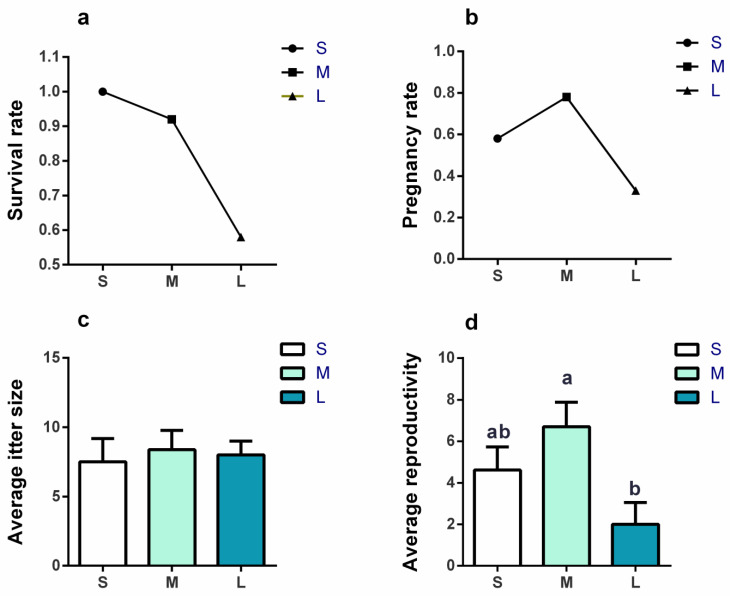
The survival rate and reproduction conditions on day 39–41. (**a**) Survival rate was not significantly affected by group size. *n* = 24 per group. (**b**) Pregnancy rate was not significantly affected by group size. *n* = 12 (S group), 9 (M group), and 12 (L group) per group. (**c**) The average litter size was not significantly affected by group size. *n* = 7 (S group), 7 (M group), and 3 (L group). (**d**) The average reproductivity was significantly affected by group size, and it was lower in the L group than that in the M group. *n* = 12 (S group), 9 (M group), and 12 (L group) per group. The average number of embryos and litters per pregnant female was defined as the average litter size. The average number of embryos and litters per female was defined as the average reproductivity per female. Survival rate and pregnancy rate were analyzed using a general linear mixed model (GLMM) with cage ID as a random factor. Average litter size and average reproductivity were analyzed by LMM. Cage ID was defined as random factor. S, M, and L are small group, medium group, and large group, respectively. Bars indicate mean ± SEM. Bars with different letters differ significantly from each other.

There were 10 and 2 voles that died in the L group and M group, respectively, which decreased the final density, but the survival rate was not significantly different among groups (*χ*^2^ = 5.132, *p* = 0.077, Figure 5a). The pregnancy rate (*χ*^2^ = 3.632, *p* = 0.163, Figure 5b) and average litter size (*F*_2,15_ = 0.383, *p* = 0.688, Figure 5c) were not significantly different among the three groups. The average reproductivity per female among the three groups was significantly different (*F*_2,30_ = 5.578, *p* = 0.009, Figure 5d), and it was 3.35-fold higher in the M group than in the L group (*p =* 0.010).

### 3.3. Effects of Initial Group Size on mRNA Expression in Male Brain

#### 3.3.1. mRNA Expression of Stress-Response Genes

**Figure 6:**
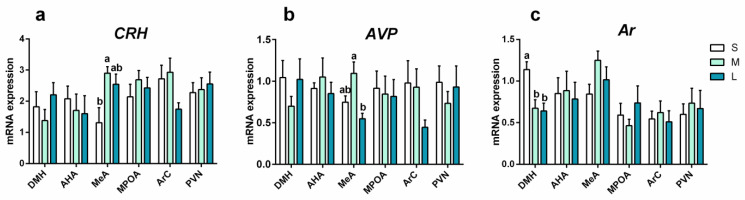
Group size affected neurochemical mRNA expression in the brain of male Brandt’s voles in a brain-region-specific manner. mRNA expression for (**a**) corticotrophin-releasing hormone (*CRH*), (**b**) arginine vasopressin (*AVP*), (**c**) androgen receptor (*Ar*). Linear mixed model (LMM) analysis was performed. Cage ID was defined as random factor. *n* = 7 (S group), 6 (M group), and 6 (L group) per group. S, M, and L are small group, medium group, and large group, respectively. Bars indicate mean ± SEM. Bars with different letters differ significantly from each other.

Group size affected mRNA expression of stress response genes in the brain of Brandt’s voles in a brain-region-specific manner. The mRNA expression of *CRH* in the MeA area was significantly different among the three groups (*F*_2,19_ = 6.126, *p* = 0.009, Figure 6a); it was 2.21-fold higher in the M group than that in the S group (*p* = 0.028).

The mRNA expression of *AVP* in the MeA area was significantly different among the three groups (*F*_2,19_ = 9.135, *p* = 0.002, Figure 6b); it was 1.99-fold higher in the M group than that in the L group (*p* = 0.018).

The mRNA expression of *Htr1a* in the Arc area was significantly different among the three groups (*F*_2,19_ = 5.680, *p* = 0.012, Figure S1a), but the multiple comparison was not significantly different.

The mRNA expression of *POMC* and *OT* in brain were all not significantly different between the three groups (Table S2).

#### 3.3.2. mRNA Expression of Reproduction-Response Genes

The mRNA expression of *GnRH* in the MeA (Figure S1b) area was different among the three groups (*F*_2,19_ = 4.261, *p* = 0.030), but did not reveal a significant difference between the three groups post hoc. The mRNA expression of GnRH in the Arc area was significantly different among the three groups (*F*_2,19_ = 3.859, *p* = 0.039), but did not reveal significant difference between the three groups post hoc.

The mRNA expression of *Ar* in the DMH area decreased significantly with the increase in group size (*F*_2,13_ = 8.15, *p* = 0.005, Figure 6c), and it was 1.69- and 1.77-fold higher in the S group than that in the M (*p* = 0.019) and L groups (*p* = 0.037).

The mRNA expression of *Rfrp* in the MeA area was significantly different among the three groups (*F*_2,19_ = 10.172, *p* = 0.001, Figure S1c), but did not reveal a significant difference between the three groups post hoc.

The mRNA expressions of *Esr1* and *Kiss1* in brain were all not significantly different between the three groups (Table S2).

### 3.4. Effects of Final Density or Group Size on mRNA Expression in Male Brain

Because some voles died due to serious fighting (2 voles and 10 voles died in the M group and L group, respectively), which did not alter the categories of group size but changed the final density, we thus analyzed the relationship between group size or final density and mRNA expression of all genes in the male brains (Table S3). We found that the final group size was significantly positively correlated with the mRNA expression of *Rfrp* in the MeA area (*r* = 0.590, *p* = 0.005), *GnRH* in the MeA area (*r* = 0.596, *p* = 0.004), and *Ar* in the MeA area (*r* = 0.481, *p* = 0.027), while it was significantly negatively correlated with the mRNA expression of *Htr1a* in the Arc area (*r* = −0.574, *p* = 0.007) and *Ar* in the DMH area (*r* = −0.438, *p* = 0.047). The final density was significantly positively correlated with the mRNA expression of *CRH* in the Arc area (*r* = 0.545, *p* = 0.011), *AVP* in the MeA area (*r* = 0.471, *p* = 0.031), and *Ar* in the DMH area (*r* = 0.565, *p* = 0.008). There was a significant negative correlation between final density and the expression of *Rfrp* in the MPOA area (*r* = −0.597, *p* = 0.004) and *Kiss1* in the MPOA (*r* = −0.457, *p* = 0.037) area.

## 4. Discussion

In this study, we found that aggression behavior was higher in the large-sized group than that in small-sized group, and male voles tended to be more anxious. These observations were consistent with our predictions. However, the mRNA expression of stress-response genes (*AVP*, *CRH*) was the highest in the initial moderate group, which was not consistent with our predictions. That was due to changes of density or group size caused by high mortality in the initial large group. The mRNA expression of stress-response genes, *AVP*, was positively correlated with the final density. We found that the average reproductivity of female voles in the initial moderate group was higher than that in the large group; the mRNA expression of *Rfrp* in the MeA was the highest in the large group, which generally supports our prediction. The mRNA expression of reproduction-response genes, *Ar*, was the highest in the small group, which did not fit our predictions. However, it was negatively correlated with group size and positively correlated with final density caused by high mortality in the initial large group.

### 4.1. Impact of Group Size on Behaviors and mRNA Expression

The stress behavior would increase with the increase in group size. Van Loo et al. [7] studied three group sizes (3, 5, 8 mice/cage) of BALB/c mice housed in a density of 80 or 125 cm^2^/mice for 14 weeks. The group with 8 mice/cage had more aggression behavior than 3 mice/cage, and larger space aggravated their aggression. In our study, the aggression behavior was more severe in the L group than in the S group, which supports our predictions (1–3) about the aggression behavior in the three groups. In open field, the mobility of voles was not affected. The anxiety level of voles among the three groups was not significantly different. This might be that the aggression behavior reduced in the end and the high mortality rate decreased the density of the L group. Female CD-1 mice (*Mus musculus domesticus*) housed individually for seven days tended to explore less and be more anxious compared with those housed with same-sex siblings (three animals per cage). However, males housed individually tended to show the opposite profile [51]. In our study, the level of anxiety in males might be higher than those in females, which is consistent with the study by Johnston and File [52]. This might be the results of competition among males for dominance. Dominant males show heavy aggression behavior and have the prior right to mate with females, gain food and water, and so on [40].

Under chronic stress, the CRH continues to be released and the HPA axis is disordered; then, the anxiety and depression behavior increase [53]. Stresses such as restraint, swimming, and attacks by predators increase the expression of c-fos in the MeA. The glucocorticoid receptor of amygdala is positively fed back to regulate the stress response, which enhances rather than inhibits the HPA response [54,55,56]. So, chronic stress increases the CRH expression in the MeA [57]. In our study, the expression of *CRH* mRNA in the MeA was the highest in the M group, which is not consistent with our prediction (2) that the mRNA expression of the stress-response gene (*CRH*) in the L group was the highest. This was caused by the decreasing aggression behavior at the end of the experiment (Figure 2c–d), because some animals died during the experiment. The mRNA expression of the *CRH* gene were measured after the change in group size and last density. This change might have buffered the social stress in the L group, then decreased the expression of the *CRH* gene. This was consistent with conventional observation that high-density crowding would increase stressful hormone levels [58].

In rodents, AVP regulates social recognition and relationship, flank marking, and aggression. Male aggression increases when injecting AVP into AH and lateral septum (LS) [59]. Huang et al. [41] found that more aggressive behavior was induced and the expression of *AVP* mRNA and protein increased in the high-density group. The *AVP*-synthesizing neurons were rich in the dorsal MeA region of rodents [60,61], and these extra-hypothalamic neurons were more common in males and androgen-dependent [62,63]. In our study, the expression of this gene in the MeA was the highest in the M group, which is not consistent with our prediction (2) that the mRNA expression of the stress-related gene (*AVP*) in the L group was the highest. Similarly, this was caused by the decreased final density in the L group buffering the high stress. It was supported by a positive correlation between the final density and mRNA expression of *AVP*. In our study, the most significant differences of stress-response genes appeared in the MeA area, which suggested that the MeA area was a main area in modulating the stress of group living.

*Htr1a* regulates stress response [64] and aggressive behavior [65]. Mice lacking *Htr1a* are more sluggish than wild mice. Their aggressive behavior reduces significantly and anxiety behavior increases significantly [66]. In our study, the mRNA expression of *Htr1a* in the Arc was negatively correlated with group size, which is not consistent with our prediction (2) that the mRNA expression of stress-response gene (*Htr1a*) in the L group was the highest, because the decreased final density and aggression in the L group at the last phase (day 35) buffered the high stress, which reduced the expression of Htr1a in the L group, and it showed a negative correlation with the final group size.

### 4.2. Impacts of Group Size on Reproduction and Gene Expression

Previous studies indicated that high density would inhibit the reproduction of animals [67]. Zhou et al. [68] found that the pregnancy rate of female Brandt’s voles in high-density areas was significantly lower than that in low-density areas, resulting in lower birth rates and slower population growth in the high-density populations. Zhang et al. [69] found that female *Microtus fortis* pair-reared in the low-density group (2 voles per cage, 90 days) were all pregnant, while the reproduction of *Microtus fortis* pair-reared in the high-density group (8 voles per cage) for 90 days or 20 days were all inhibited and no females were pregnant. When transferring them to low-density for 20 days, there were 15% and 50% pregnancy rates, respectively. The average litter size of the high-density group was lower than that of the low-density group. In our study, we found that the pregnancy rate of female voles was not significantly different among groups, but it showed a tendency to be highest in the M group. The average reproductivity per female in the M group was higher than that in the L group, which was consistent with our prediction (1) that the reproduction performance of voles in the M group is the highest. Male reproductive success is always decided by the number of female partners; however, female reproductive success is not obviously acquired by mating with multiple males, because it is decided by the number of ova or offspring [70]. Too small a group size would decrease the reproductive performance of animals, because females often need more mates to stimulate ovulation [71,72,73]. Meanwhile, multiple mating increases female fitness, as females produce more offspring or improve the quality of offspring [74,75]. So, one spouse resulted in the lower reproduction performance of the S group. However, in the large-sized group, serious fighting for mates also reduced the reproduction of females, like the observed low reproductivity in the L group. In addition, the wrong identification of sex induced that the sex ratio (♀/♂) in the M group was the highest (1:2) among the three groups, but it did not affect the reproduction performance, because the sex ratio was not correlated with reproduction performance (data not shown).

Stress often inhibits reproduction, because stress can induce a series of changes in physiological functions [76,77,78,79]. The relationship between reproductive gene expression and group size has been rarely investigated. GnRH neuronal network produces pulsed and surge patterns of gonadotropin secretion, which are essential for puberty and fertility. Ar signaling participates in the development of male-specific phenotypes during embryogenesis, spermatogenesis, sexual behavior, and fertility during adult life [80]. Early social stress makes male guinea pigs express few Ars in the MPOA and Arc compared with unexperienced control males [33]. In our study, the observation on mRNA expression of *Ar* gene in the DMH area does not support our prediction (1) that the mRNA expression of reproduction-response genes (*Ar*) was the highest in the M group. However, it was the lowest in the L group, which demonstrated that androgen production was inhibited under high stress, so its feedback was reduced. DMH plays a role in regulating neuroendocrine, autonomic, and somatic responses to external stimuli sensory feedback and cognitive/motivational input [81]. Male Syrian hamsters whose DMH were injured did not present seasonal reproductive quiescence, which may be caused by disappearing melatonin-binding sites in the DMH and increasing the feedback sensitivity of gonadotropins to steroid hormones [82,83]. In our study, the mRNA expression of the *Ar* gene in the DMH area indicated that male voles in the L group would have low fertility.

Stress induces an increase in *Rfrp*, which affects neural circuits about pairing and pregnancy. *Rfrp*-expression cells in the DMH area of nonreproductive males were higher than those of reproductive males, which was associated with reproductive quiescence in subordinate males [84]. Previous studies showed that MeA played a role in regulating male sexual arousal. Males that received flutamide in MeA exhibited fewer noncontact penile erections and longer latencies before their first sexual behavior [85]. In our study, the expression of this gene in the MeA was the highest in the L group, which is consistent with our prediction (2) that the mRNA expression of the reproduction-related gene (*Rfrp*) in the L group was the highest. This was further supported by the significant and positive correlation between the mRNA expression of *Rfrp* in the MeA and the final group size. These results suggested that reproduction of male voles in the L group was largely inhibited. The categories of S, M, and L groups were not changed by the dead voles; thus, if no voles died, the difference in reproduction should not be changed, but the stress-response RNA expression should be larger or largest in the L group.

### 4.3. Limitation of the Study

There are several limitations of this study. First, to minimize the number of voles used, we had fewer replicates of the L group. Second, due to unexpected death of some voles, which altered the final density and group size of some replicates, the stress-response behavior and mRNA expression showed a mismatch with the initial designed group size. Therefore, the results of this study should be carefully interpreted on the L group, and need to be verified in future studies. To overcome these two defects, it is necessary to increase replicates of the large-sized group. Finally, in this study, we only analyzed the gene expression of males. The mRNA expression of females needs to be investigated in future studies, but the estrus cycles of females should be taken into account.

## 5. Conclusions

In our study, Brandt’s voles were housed in different-sized groups for 35 days. We found that voles in the large group suffered from the highest aggression behaviors, male voles were more anxious, and the reproductivity per female in the moderate group was the highest. Stress- or reproduction-response gene expressions were more driven by the final group size and final density: stress-response gene expression increased with the increase in final group size or density, while the reproduction-response gene, *Rfrp*, expression increased with final group size. Our results suggested that a moderate group size is beneficial to the fitness of voles, which is driven by both stress- and reproduction-response gene expression. Considering the limitation of this study, future research should be directed to test these observations with more replicates or in natural or seminatural conditions.

## Figures and Tables

**Table 1:** The primers of all genes and their amplified products.

Genes	Primers		Products (bps)
(A) Stress-response genes			
Corticotropin-releasing hormone (*CRH*)	F	5′ GCAAATGCTGCGTGCTTTC 3′	231
	R	5′ TCTCTTCTCCTCCCTTGGTAG 3′	
Pro-opiomelanocortin-alpha (*POMC*)	F	5′ TTGGAAAGATAGCGGGAGAG 3′	187
	R	5′ GCAGAGGCAAACAAGATTGG 3′	
Arginine vasopressin (AVP)	F	5′ ACGCTCTCCGCTTGTTTC 3′	110
	R	5′ CACTGTCTCAGCTCCATGTC 3′	
Oxytocin (*OT*)	F	5′ TGCCAGGAGGAGAACTACC 3′	149
	R	5′ TCCGAGAAGGCAGACTCAG 3′	
5-hydroxytryptamine receptor1A (*Htr1a*)	F	5′ GTTGGACAGCGACAAAGTG 3′	212
	R	5′ TGGAGCGGGAAGTTCTTAC 3′	
(B) Reproduction-response genes			
Gonadotropin-releasing hormone 1 (*GnRH*)	F	5′ CGGCATTCTACTGCTGACTG 3′	239
	R	5′ GCCTGGCTTCCTCTTCAATC 3′	
*Kiss1*	F	5′ AAGGAATCGCGGTATGCAG 3′	203
	R	5′ CCGAAGGAGTTCCAGTTGTAG 3′	
RFamide-related peptides (*Rfrp*)	F	5′ CAAGACACCCGCTGATTTGC 3′	113
	R	5′ TTCGCTTTCCACCAGGACTC 3′	
Estrogen receptor 1 (alpha) (*Esr1*)	F	5′ AATGACTATGCCTCTGGCTACC 3′	184
	R	5′ TGCCCACTTCGTAACACTTG 3′	
Androgen receptor (*Ar*)	F	5′ CAGAGGCAAAGTCTAAAGC 3′	125
	R	5′ CAACTATGGTGGAGATTCG 3′	
(C) Reference gene			
Glyceraldehyde-3-phosphate dehydrogenase (*Gapdh*)	F	5′ ATCACTGCCACCCAGAAG 3′	191
	R	5′ TCCACGACGGACACATTG 3′	

(A) The stress-response genes included *CRH*, *POMC*, *AVP*, *OT*, and *Htr1a* genes; (B) the reproduction-response genes included *GnRH*, *Kiss1*, *Rfrp*, *Esr1*, and *Ar* genes; (C) the reference gene was the *Gapdh* gene.

## Data Availability

The datasets are available from the corresponding author on reasonable request.

## Supplementary Materials

The following supporting information can be downloaded at https://www.mdpi.com/article/10.3390/brainsci13020311/s1, Table S1: The final sample size in the three groups; Table S2: Impacts of group size on mRNA expression of Brandt’s voles (no significance); Table S3: The Spearman correlation between the final group size and mRNA expression of all genes in male brains, and the relationship between the final density and mRNA expression of all genes in male brains; Figure S1: Group size affected neurochemical mRNA expression in the brains of male Brandt’s voles in a brain-region-specific manner (no significant differences in post hoc tests).
