# Supplementary material for: Effects of Group Size on Behavior, Reproduction, and mRNA Expression in Brains of Brandt’s Voles
Source: Brain Sci. 2023 Feb 12;13(2):311. doi: 10.3390/brainsci13020311 (PMC9954483; doi:10.3390/brainsci13020311)
Supplement: Supplementary file 1 [file brainsci-13-00311-s001.zip › brainsci-2051375-supplementary.pdf]

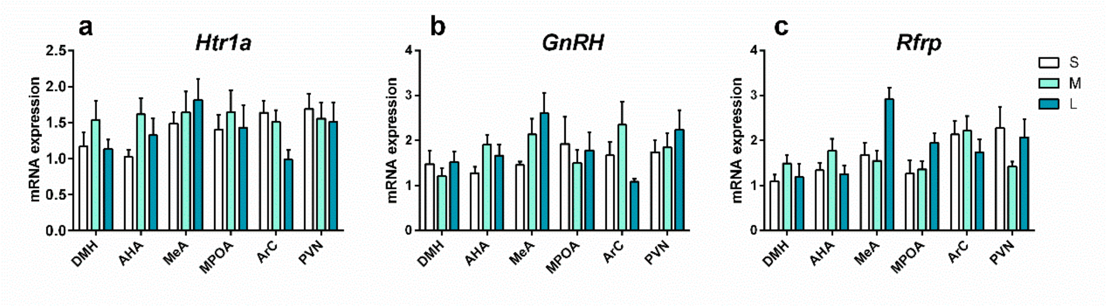

Figure S1. Group size affected neurochemical mRNA expression in the brains of male Brandt's voles in a brain-region-specific manner (no significant differences in post hoc tests). Figure S1a) The mRNA expression of *Htr1a* in the Arc area was significantly different among the three groups, but the multiple comparison was not significantly different. Figure S1b) The mRNA expression of *GnRH* in the MeA area was different among the three groups, but did not reveal a significant difference between the three groups post hoc. The mRNA expression of GnRH in the Arc area was significantly different among the three groups, but did not reveal significant difference between the three groups post hoc. Figure S1c) The mRNA expression of *Rfrp* in the MeA area was significantly different among the three groups, but did not reveal a significant difference between the three groups post hoc.

Table S1. The final sample size in the three groups

Group	Replicate code	Number of Males	Number of Females	Total number
Small-sized group	1	1	1	2
	2	1	1	2
	3	1	1	2
	4	1	1	2
	5	1	1	2
	6	1	1	2
	7	1	1	2
	8	1	1	2
	9	1	1	2
	10	1	1	2
	11	1	1	2
	12	1	1	2
Medium-sized group	1	1	1	2
	2	2	2	4
	3	3	1	4
	4	4	0	4
	5	3	1	4
	6	2	2	4
Large-sized group	1	2	3	5
	2	1	1	2
	3	4	3	7

Table S2. Impacts of group size on mRNA expression of Brandt's voles (No significance)

Brain area	Gene	$F_{2,19}$	P
DMH	*POMC*	3.264	0.089
	OT	0.211	0.812
	Esr1	1.657	0.217
	Kiss1	0.563	0.613
AHA	*POMC*	0.133	0.876
	OT	1.170	0.332
	Esr1	0.374	0.693
	Kiss1	0.943	0.407
MeA	*POMC*	1.238	0.312
	OT	0.264	0.771
	Esr1	1.535	0.241
	Kiss1	0.749	0.507
MPOA	*POMC*	0.135	0.876
	OT	0.485	0.632
	Esr1	0.949	0.405
	Kiss1	1.546	0.263
Arc	*POMC*	1.158	0.335
	OT	0.277	0.761
	Esr1	0.377	0.695

	Kiss1	2.364	0.121
PVN	*POMC*	1.822	0.189
	OT	0.342	0.718
	Esr1	1.451	0.259
	Kiss1	1.570	0.234

Table S3. The Spearman correlation between the final group size and mRNA expression of all genes in male brains, and the relationship between the final density and mRNA expression of all genes in male brains.

Genes	Final group size	Final Density
CRH	NS	Arc + (*r*=0.545, *P*=0.011）
POMC	NS	NS
AVP	NS	MeA + (*r*=0.471, *P*=0.031)
OT	NS	NS
Htr1a	Arc - (*r*=-0.574, *P*=0.007)	NS
GnRH	MeA + (*r*=0.596, *P*=0.004)	NS
Kiss1	NS	MPOA - (*r*=-0.457, *P*=0.037)
Rfrp	MeA + (*r*=0.590, *P*=0.005)	MPOA - (*r*=-0.597, *P*=0.004)
Ar	DMH - (*r*=-0.438, *P*=0.047)<br>MeA + (*r*=0.481, *P*=0.027)	DMH + (*r*=0.565, *P*=0.008）
Esr1	NS	NS

NS means there is no significance.
